# Perceived discrimination among older adults living in urban and rural areas in Brazil: a national study (ELSI-Brazil)

**DOI:** 10.1186/s12877-019-1076-4

**Published:** 2019-03-04

**Authors:** Luciana de Souza Braga, Waleska Teixeira Caiaffa, Ana Paula Romanelli Ceolin, Fabíola Bof de Andrade, Maria Fernanda Lima-Costa

**Affiliations:** 1 Observatório de Saúde Urbana de Belo Horizonte, Faculdade de Medicina, Universidade Federal de Minas Gerais, Avenida Professor Alfredo Balena, n° 190, Belo Horizonte, Minas Gerais 30130-100 Brazil; 2 Instituto René Rachou, Fundação Oswaldo Cruz, Avenida Augusto de Lima, n°1715, Belo Horizonte, 30190-002 Brazil

**Keywords:** Discrimination, Urban population, Rural population, Socioeconomic factors

## Abstract

**Background:**

Research on discrimination and health focused on older adults has been scarce, comparatively with younger and middle-aged adults. Considering where people live matters, accurate measures of perceived discrimination might consider how the place of residence interferes on discriminatory experiences. This study aimed to assess the association between perceived discrimination and urban/rural place of residence among a representative sample of older adults in Brazil.

**Methods:**

Data came from the baseline of the Brazilian Longitudinal Study of Aging (ELSI-Brazil), conducted in 2015/2016, with individuals aged 50 years and older. Perceived Discrimination was measured by means of the following question: “*In the past 12 months have you felt a victim of any type of discrimination*” with five possible answers: (1)“*when you sought medical services or health care*?”, (2)“*in social gatherings*?”, (3)“*in the work place?*”, (4)“*within the family?*”, (5)“*due to where you live?*”. Participants who answered yes for any of the five domains were coded as having reported an experience of discrimination. The main exposure variable was the urban-rural classification of the households, carried out according to the methods employed by the Brazilian Institute of Geography and Statistics during the 2010 Population Census. Other covariates included: age, sex, skin color, household wealth and education. Multiple Poisson regression was used to estimate prevalence ratios and their respective 95% confidence interval for the association between discrimination and independent variables.

**Results:**

Prevalence of any perceived discrimination among Brazilian older adults was 16.8%. Regardless the place of residence (either urban or rural), participants reported health care settings as the most common domain where discriminatory experiences occurred and the work place as the least common. According to the adjusted model, perceived discrimination was significantly higher among urban dwellers when compared to their rural counterparts, independent of sociodemographic characteristics, health status and neighborhood social environment. The outcome was significant associated with skin color, education and health status.

**Conclusions:**

Urban environment plays a core role in perceived discrimination and health care settings constitute the most common domain where discriminatory experiences occurred. Our findings may contribute to fulfill the knowledge gap on discrimination among older adults living in developing countries.

**Electronic supplementary material:**

The online version of this article (10.1186/s12877-019-1076-4) contains supplementary material, which is available to authorized users.

## Background

Perceived discrimination has been defined as an individual’s perception of being treatedunfairly by other people due to some personal attribute, such as race, ethnicity, age, gender, socioeconomic status, weight, sexual orientation, or other characteristics [1, 2]. Unfair treatment usually involves discriminatory practices of dominant groups to maintain privileges they accrue through subordinating groups they oppress, which typically revolve around notions of innate superiority and inferiority, difference, or deviance [3, 4].

A large and growing body of research suggests that self-reported experiences of discrimination are a form of psychological stress that has an adverse impact on both mental and physical health outcomes, across multiple population groups in a wider range of cultural and national contexts [4–6]. Researchers have found associations between reports of discrimination and distress, clinically diagnosed mental disorders (e.g., generalized anxiety, posttraumatic stress disorder, depression), all-cause mortality and a variety of objective clinical disease outcomes such as preclinical endpoints - nighttime blood pressure and visceral fat - and silent indicators of premature aging - higher allostatic load and increased oxidative stress [5, 6]. Regardless of effects on health, unjustly denying people fair treatment constrain possibilities for living fully expressed and create patterns of health inequities [3, 4]. Thus, perceived discrimination’ studies may contribute to identify what drives population patterns of health and health inequities and to generate knowledge useful for guiding policies and actions to tackle inequities.

Research on discrimination and health focused on older adults has been scarce, comparatively with younger and middle-aged adults. In general, researchers suggest that older adults who internalize negative attitudes towards themselves are at increased risk for functional [7] and cognitive decline [8], life dissatisfaction [1, 9], depression [1], besides social withdrawal [9, 10], reduction in cultural engagement and reluctance to visit health professionals [10]. Among older adults who live in developing countries, literature is even scarcer. The studies usually represent major urban centers, focus primarily on racial discrimination and older adults just integrate larger samples composed by younger participants rather than constitute the target population under analysis [11–13].

In Brazil, evidences from a large metropolitan region showed that around 9% of the respondents reported some type of discrimination, higher among black, women and lower among those aged 60 years and over [11]. Additional associated factors included poorer health status and low social trust [11]. Data from a larger survey of civil servants showed that among individuals aged 40 and older, black were more likely to report lifetime discrimination, particularly black men and for most race-by-gender groups, perceived discrimination increased with educational attainment [13].

Brazil has rapidly transitioned from a low-income country, primarily rural in the mid-1950s, to one of the top ten economies in the world, with 84% of the population living in urban areas [14]. The latest Brazilian census indicated that nearly 33 million people aged 50 and older lived in cities, and about 6 million were rural dwellers [15]. The magnitude of rural Brazil is not negligible and these areas still play a fundamental role in keeping economics, social cohesion and environmental sustainability [16, 17]. In its turn, urban dwellers may experience advantages and disadvantages [18, 19], such as many adverse health outcomes and large health inequalities [20]. It already became apparent that urban disadvantaged areas may have similar if not worse health outcomes than rural areas [18, 21]. Some authors argue that discrimination would manifest strongly in areas with higher levels of social inequalities [3]. If where people live matters, accurate measures of perceived discrimination might consider how the place of residence affects discriminatory experiences.

Using data from the Brazilian Longitudinal Study of Aging (ELSI-Brazil) baseline survey, we examined the cross-sectional association between perceived discrimination and urban/rural place of residence, among older adults. Our objective is also to identify the main individual characteristics related to higher reports of discriminatory experiences, fostering better understanding of the distribution, magnitude, and interrelationships among risk factors for exposure to discrimination. Considering discrimination may manifest strongly in areas with higher levels of social inequalities, we hypothesized that urban residence would be positively associated with perceived discrimination, independent of sociodemographic characteristics. Given the marginalized racial status of blacks in Brazil and the evidences from abovementioned studies, we also expected higher reports of discriminatory experiences within this group.

## Methods

### Study population

ELSI-Brazil is a nationally representative population-based cohort study of non-institutionalized people aged 50 years and older residing in 70 municipalities across the 5 great Brazilian regions [22]. The baseline survey was conducted between 2015 and 2016. To ensure that the sample represents the urban and rural areas of the small, medium and large municipalities, the ELSI-Brazil sampling used a design with selection stages, combining stratification of primary sampling units (municipalities), census tracts and households. The analytic sample for this study included all ELSI-Brazil participants (*n* = 9412). Individuals were excluded if they had missing data on perceived discrimination and place of residence. The resulting analytic sample was comprised of 9383 participants. Detailed information on design, methods of recruitment and covered topics is available elsewhere [22]. ELSI-Brazil was approved by the Ethics Board of Oswaldo Cruz Foundation (FIOCRUZ), Minas Gerais (CAAE: 34649814.3.0000.5091). All participants signed separate informed consent forms in advance of their participation in the study.

### Measures

#### Perceived discrimination

To our dependent variable, collected information was based on the question: “*In the past 12 months have you felt a victim of any type of discrimination*”, that was followed up with five possible domains: (1)“*when you sought medical services or health care*?”, (2)“*in social gatherings*?”, (3)“*in the work place?*”, (4)“*within the family?*”, (5)“*due to where you live?*”. For each domain, participants who answered “yes” were coded as having reported an experience of discrimination. An indicator variable, termed “any discrimination”, was developed to capture whether the respondents reported at least one episode of discrimination in any above-mentioned domain.

#### Place of residence

The main exposure variable was the urban-rural classification of the households, carried out according to the methods employed by the Brazilian Institute of Geography and Statistics (IBGE) during the 2010 Population Census [23]. Firstly, IBGE defines the urban-rural classification of each census tract from administrative limits set out by local laws [23]. Within each municipality boundaries, these local laws determine an imaginary line called “urban perimeter”. The census tracts located within the “urban perimeter” are termed urban while the residual areas are designated rural. Second, IBGE assigns to the households the same urban-rural classification of the census tract to which these residences belong. Because the sampling design of ELSI-Brasil took into account the urban-rural classification of each census tract, the households’ classification was already known previously to the interview.

#### Covariates

Individual sociodemographic characteristics included age (50–59 years, 60–69 years, 70–79 years, > 80 years), sex (male or female), ethno racial self-classification, household wealth and educational attainment (less than 4, 4–7, and 8 or more years). ELSI-Brazil participants self-declared race according to one of the following IBGE categories: black, brown, white, yellow (Asian) and indigenous. The latter two categories constitute nearly 1% of the Brazilian population over 50 years old [15]. Thus, considering the small sample size of both yellow and indigenous participants, these categories were grouped for analytic purposes, regardless of the acknowledged cultural differences between them. Analyses showed similar trends in terms of direction and magnitude when groups were treated separately (data not shown). The household wealth was assessed through data on ownership of household durable assets, such as home appliances and vehicles, and domestic employees. Based on principal components analysis (PCA), a score of socioeconomic position was generated and participants were divided into quartiles, from the ‘most poor’ to the ‘most wealthy’ [24]. A binary variable whereas the 3 lowest quartiles versus the top quartile was created and employed during the analyses.

Finally, we also included health status and neighborhood social environment indicators whereas both could confound or partially mediate the association between perceived discrimination and place of residence. Due to collinearity among health indicators, a composite measure of the health status based on PCA was obtained (see Additional file 1). The selected variables included self-rated health, number of the last 30 days spent in poor mental and/or physical health, and history of medical diagnosis from a list of 12 prevalent chronic diseases/conditions, such as hypertension, diabetes, depression and arthritis. The resulting health status score was divided into tertiles and the lowest one, termed “few health problems”, represents the reference group. The neighborhood social environment was assessed by the question: “*Do you believe you can trust most people in your neighborhood*?” Responses were coded as low, medium or high social trust. All covariates rely on self-report.

### Statistical analysis

We calculated age-and gender-adjusted prevalence rates for each study variable according to the place of residence and estimated perceived discrimination prevalence by sex, age, ethno racial self-classification and educational attainment using Poisson regression. To verify differences among categories, we used the proportion test, considering a level of significance of 0.05. Use of Poisson models was justified because our outcomes were relatively common (prevalence > 10%), especially the main one (any discrimination) [25]. To verify the univariate and multivariate associations between “any discrimination” and the independent variables, we fit Poisson regression to estimate the prevalence ratios (PR) and their respective 95% confidence interval (95% CI). The multivariate model was tested including all independent variables. To examine whether the place of residence had a different effect on race, we include an interaction term. Those variables with a *p*-value > 0.05 were removed. From the multivariate model, we plotted the predicted probabilities of reporting any discrimination.

Data analysis was performed using Stata 12.0 statistical program [26]. Through the svy command, we took into account the sample design, individual weights and aggregation.

## Results

Among older adults, nearly 85% live in urban areas and 15% are rural dwellers, a proportion quite similar to the patterns of the 2010 Brazilian Population Census. Table 1 shows that rural dwellers present a significant higher proportion of blacks (11.9% versus 9.3% in urban areas), and browns (49.7% versus 43.8% in urban areas), worse educational attainment (89.0% have less than 8 years of schooling versus 59.8% in urban areas) and a higher proportion of individuals belonging to the lowest household wealth quartiles (94.2% versus 71.5% in urban areas). Otherwise, low social trust was predominant among older adults who lived in urban areas (19.1% versus 15.0% in rural areas). The age and sex-adjusted prevalence of perceived discrimination (any) among Brazilian older adults was 16.8%, higher among urban dwellers for all domains. Regardless the place of residence, participants reported health care settings as the most common domain where discriminatory experiences occurred.

**Table 1:** Age and sex-adjusted prevalence of descriptive statistics, by place of residence (ELSI-Brazil/2015–2016)

	Urban	Rural	Total
N (unweighted)	7912	1471	9383
% (weighted)	84.7	15.3	100.0
Aged 50–59 years^1^	48.0	46.0	47.7
60–69 years^1^	29.4	30.9	29.6
70–79 years^1^	15.4	16.9	15.6
80+ years^1^	7.2	6.2	7.1
Female^1^	53.9	54.3	53.9
Male^1^	46.2	45.7	46.1
White*	44.1	35.2	42.7
Black*	9.3	11.9	9.7
Brown*	43.8	49.7	44.7
Yellow	1.1	1.0	1.1
Indigenous	1.8	2.3	1.9
Educational attainment (< 4 years)*	28.5	56.8	32.8
4–7 years	31.3	32.2	31.4
8+ years*	40.2	10.8	35.7
Lowest household wealth (3 lowest quartiles)*	71.5	94.2	75.0
Highest household wealth (top quartile)*	28.5	5.8	25.0
Few health problems^2^	35.6	36.1	35.7
Some health problems^2^	32.3	31.9	32.2
Many health problems^2^	32.0	32.1	32.0
Low social trust*	19.1	15.0	18.5
Medium social trust*	26.3	30.0	26.9
High social trust	54.6	54.9	54.6
Perceived discrimination (any)*	17.6	12.2	16.8
In seeking health care*	11.4	8.7	11.0
In social gatherings	3.3	2.5	3.1
In the work place*	2.7	1.1	2.5
Within the family*	3.8	2.6	3.6
Due to where live *	3.7	2.3	3.5

^1^=Non-adjusted. * = significant difference between urban and rural (p < 0.05). ^2^Composite measure obtained through principal components analysis, from the following variables: self-rated health, number of the last 30 days spent in poor mental and/or physical health, and history of medical diagnosis from a list of 12 prevalent chronic diseases/conditions. Results took into account complex sample design and sample weights

Table 2 identifies the relationship between reports of discriminatory experiences and sociodemographic characteristics. Except for perceived discrimination within the family, no male/female trend was found regarding perceived discrimination. Age showed an inverse gradient with reports of discriminatory experiences, for all analyzed domains. Whereas ethno racial self-classification, yellow and indigenous reported higher discrimination (any, when sought health services and due to where live) than any other race category. Regarding educational attainment, the most educated individuals were more likely to report any discrimination, but only significant for social gatherings and work place domains.

**Table 2:** Perceived discrimination by sociodemographic characteristics (ELSI-Brazil/2015–2016)

	Sex (n)		Age (years) (n)				Ethno racial self-classification (n)				Educational attainment (years) (n)		
	Female (5314)	Male (4098)	50–59 (3980)	60–69 (2875)	70–79 (1781)	80+ (776)	White (3590)	Black (887)	Brown (4283)	Yellow/Indigenous (310)	< 4 (3463)	4–7 (2845)	8+ (3042)
	%		%				%				%		
Perceived discrimination (any)	17.6	15.8	18.5	17.5	13.6	8.8*	14.3	21.9	17.7	26.2*	14.4	17.9	18.0*
In seeking health care	11.4	10.5	11.5	11.8	9.9	6.4*	9.2	13.9	11.8	17.3*	10.5	11.7	10.9
In social gatherings	3.2	3.1	3.6	3.2	2.7	0.8*	2.1	6.2	3.3	5.0*	2.7	2.7	3.9*
In the work place	2.3	2.7	3.6	2.1	0.7	0.2*	2.0	3.0	2.6	4.8	1.3	2.4	3.6*
Within the family	4.6	2.5*	3.8	3.9	3.5	2.0	3.4	3.3	3.8	5.1	3.5	4.1	3.3
Due to where live	3.8	3.2	3.8	4.0	3.0	0.8*	2.6	4.8	4.0	7.0*	3.2	3.5	4.0

* = Significant difference (*p* < 0.05)

Table 3 presents the results of our fitted model, which presented a good fit (Deviance Goodness of Fit = 4757.694). It shows that perceived discrimination is 34% higher among urban dwellers when compared to their rural counterparts, independent of sociodemographic characteristics, health status and neighborhood social environment. Among older adults, perceived discrimination was positively associated to ethno racial self-classification, higher educational attainment (PR = 1.17 to individuals with 4–7 years of schooling; PR = 1.24 to those with > 8 years of schooling) and worse health status (PR = 1.65 to individuals with some health problems; PR = 2.40 to those with many health problems). Age and social trust showed an inverse association with perceived discrimination. Sex and household wealth showed no association with perceived discrimination. Once the interaction term between place of residence and ethno racial self-classification was insignificant, it was not included in the final model.

**Table 3:** Factors associated to perceived discrimination among older adults (ELSI-Brazil/2015–2016)

		Unadjusted^2^		Adjusted^3^	
	N^1^	PR	95% CI	PR	95%CI
Place of residence (reference: rural)	192				
Urban	1425	1.44	(1.13–1.84)	1.34	(1.06–1.69)
Aged (reference: 50–59 years)	787				
60–69 years	491	0.95	(0.84–1.07)	1.00	(0.88–1.14)
70–79 years	257	0.74	(0.62–0.88)	0.82	(0.68–1.00)
80+ years	82	0.48	(0.35–0.65)	0.52	(0.38–0.72)
Sex (reference: female)	950				
Male	667	0.89	(0.79–1.01)	1.08	(0.94–1.24)
Ethno racial self-classification (reference: white)	526				
Black	189	1.54	(1.25–1.89)	1.49	(1.25–1.78)
Brown	772	1.24	(1.04–1.48)	1.20	(1.03–1.41)
Yellow / Indigenous	77	1.84	(1.41–2.40)	1.57	(1.16–2.14)
Educational attainment (reference: < 4 years)	523				
4–7 years	504	1.24	(1.10–1.40)	1.17	(1.03–1.32)
8+ years	579	1.25	(1.06–1.47)	1.24	(1.05–1.46)
Household wealth (reference: 3 lowest quartile)	1302				
Highest household wealth (top quartile)	304	0.86	(0.73–1.02)	0.87	(0.74–1.03)
Health status (reference: few health problems)^4^	338				
Some health problems	540	1.66	(1.40–1.97)	1.65	(1.39–1.96)
Many health problems	739	2.46	(2.09–2.89)	2.40	(2.02–2.85)
Neighborhood social environment (reference: low social trust)	495				
Medium social trust	479	0.75	(0.65–0.86)	0.77	(0.66–0.89)
High social trust	629	0.49	(0.42–0.56)	0.54	(0.47–0.62)

PR^:^ prevalence ratio; 95% CI: 95% confidence interval. ^1^Number of participants who reported perceived discrimination. ^2^Univariate analyses; ^3^Multivariate analyses. ^4^Composite measure obtained through principal components analysis, from the following variables: self-rated health, number of the last 30 days spent in poor mental and/or physical health, and history of medical diagnosis from a list of 12 prevalent chronic diseases/conditions. Results took into account complex sample design and sample weights

**Fig. 1:**
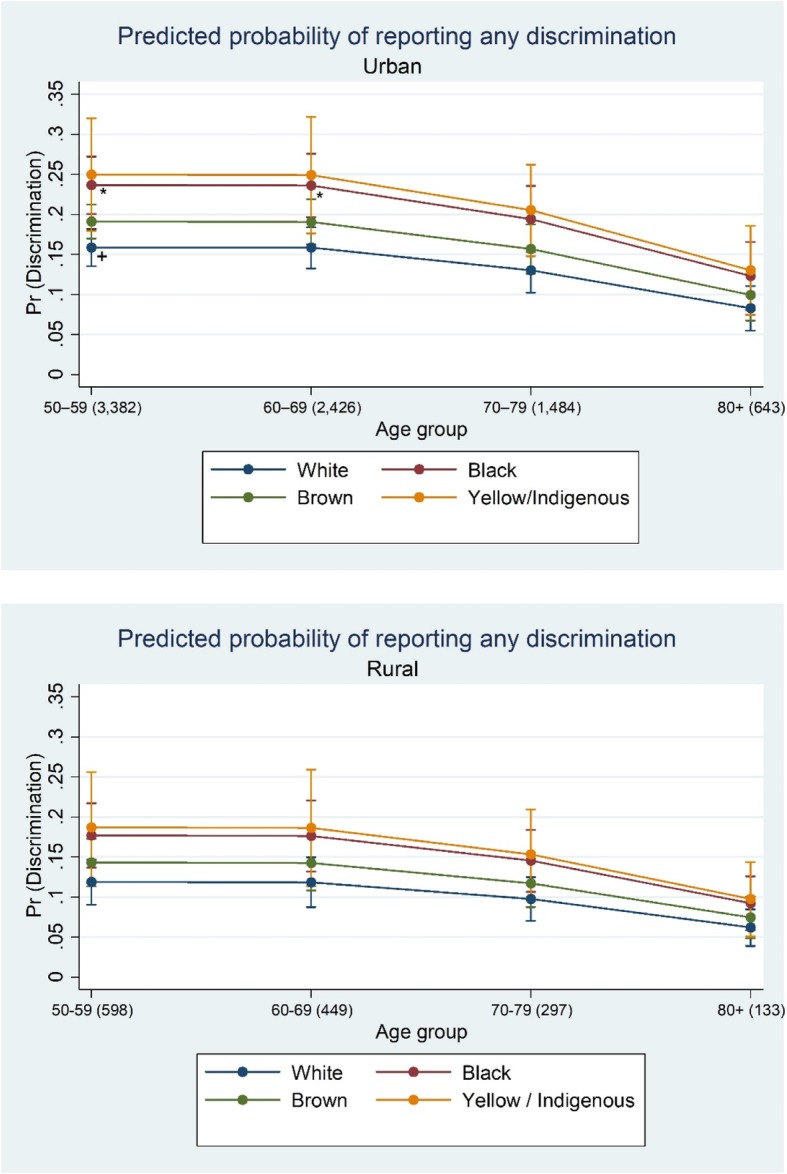
Predicted probabilities of reporting any discrimination by age group, ethno racial self-classification, and place of residence (ELSI-Brazil/2015–2016). Notes: Age group (sample size). Predicted probabilities obtained from Poisson regression controlling for age, sex, ethno racial self-classification, educational attainment, wealth, health status, and neighborhood social environment. *Difference is statistically significant from reference category (+) at the *p* < 0.05 level

**Fig. 2:**
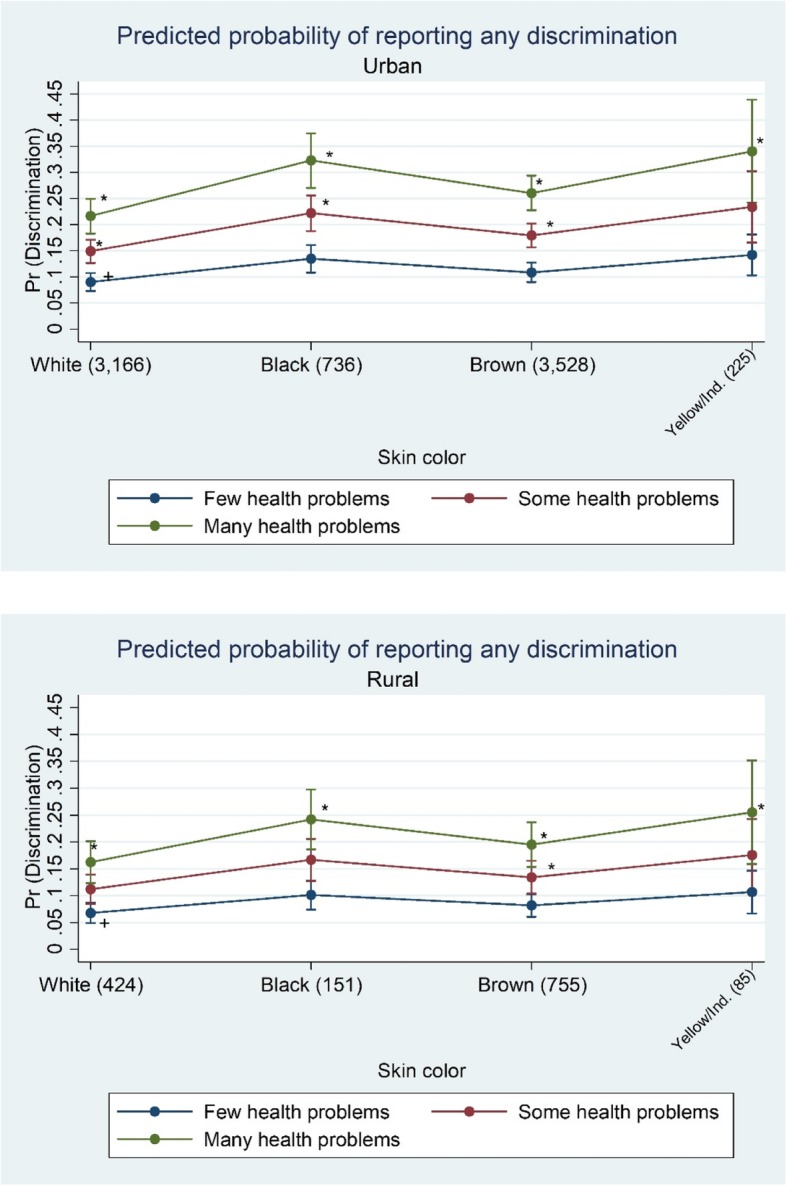
Predicted probabilities of reporting any discrimination by health status, ethno racial self-classification, and place of residence (ELSI-Brazil/2015–2016). Notes: Yellow/Ind.: yellow/indigenous. Skin color (sample size). Predicted probabilities obtained from Poisson regression controlling for age, sex, ethno racial self-classification, educational attainment, wealth, health status, and neighborhood social environment. *Difference is statistically significant from reference category (+) at the *p* < 0.05 level

Finally, the figures show how the predicted probabilities of reporting any discrimination varied by place of residence, skin color, and age group (Fig. 1) or health status (Fig. 2). Discrimination reports were highest among urban dwellers, regardless ethno racial self-classification, age group or health status. Despite these important magnitude differences between urban and rural dwellers, trends were quite similar for both areas: (1) participants who identify as yellow or indigenous were the worse-off group, followed by blacks and browns, at all ages and for every health status groups; (2) predicted probabilities decline after age 60–69 and become narrow at age 80 and over; (3) those who reported more health problems constituted the worse-off groups, regardless ethno racial self-classification.

## Discussion

In the first representative epidemiologic investigation of perceived discrimination among older adults in Brazil, we found that around 17% reported a discriminatory experience (any) in the previous year. Furthermore, we also found independent effects of place of residence on perceived discrimination, even after adjusting for socioeconomic status, health status and neighborhood social environment. Older adults living in urban areas reported 34% more discrimination than their rural counterparts. This finding supports our initial hypothesis that place of residence matters and urban residence is positively associated with perceived discrimination, independent of other characteristics. Moreover, our results also show that yellow and indigenous older adults were more likely to report discrimination, while blacks occupied the second worse-off position, followed by browns. It constitutes an unexpected finding, once we first hypothesized higher reports of discriminatory experiences among blacks.

Regarding perceived discrimination prevalence, our findings differs from both international and national literature. Brazilian older adults reported a lower prevalence of perceived any discrimination when compared to their English (39.3%) and Americans counterparts (over 60%) [2, 27]. These differences might be partially attributed to variability in the way discrimination was measured, along with possible cultural differences. Both above-mentioned studies assessed discrimination more broadly, based on the frequency with which five everyday discriminatory events had happened. Even though these scales are quite comparable to ELSI’s scale, once they measure if individuals were treated disrespectfully or received poorer quality services in day-to-day life situations, they keep slight differences. In addition, some authors argue that the persistent inequality in Brazil (Gini coefficient = 0.51 in 2015) might contribute to some individuals interpret discriminatory experiences as normal situations and do not report them [3, 28]. Social inequalities in Brazil are still remarkable and higher than United States (Gini coefficient = 0.41 in 2016) and England (Gini coefficient = 0,33 in 2015) [28]. Considering national data, our results showed an 8% higher prevalence of perceived discrimination [11]. However, this previous study also included young and middle adults, all from a specific metropolitan region in Brazil, which might contribute to justify the differences found.

Regarding the place of residence, rural dwellers were less likely to acknowledge discrimination. It is possible that older adults living in rural areas encounter less discriminatory experiences than their counterparts who live in cities. In this case, rural dwellers would report lower levels of perceived discrimination and urban living might be understood as an exposure factor to discriminatory experiences. The core aspects and cultural values that define the rural ways of life in Brazil might contribute to these findings. Characteristics such as smaller populations and low demographic density, marked by less degree of social differentiation, stratification and complexity, where closer and solidarity relationships among neighbors prevail [16], might favor low levels of discrimination occurrence. An alternative explanation for the higher levels of discrimination found in urban areas is that urban older adults are more aware of discrimination and therefore more readily to report it, or are more likely to label an experience as due to discrimination. Thus, the place of residence affects the tendency to perceive and/or report discriminatory treatment. An additional aspect of interesting regarding urban-rural differences is that no differences on health status were found. Even though urban-living in disadvantaged areas have similar if not worse health outcomes than rural areas, [18, 21] rural living in Brazil is often related to important restrictions on access to goods, services and opportunities [16, 29]. Further studies are necessary to better investigate this finding among older Brazilian. Additionally, considering the nested nature of our data, multilevel analysis might help us to improve our understanding on how the place of residence contributes to perceived discrimination.

Adjustment for socioeconomic status, health status and neighborhood social environment in the current study slightly reduced the relation of place of residence to perceived discrimination, suggesting that a part of this relation may be mediated by these factors. On age, as it increased, participants reported lower levels of discrimination and a significant negative association was observed for older old. Our findings are consistent with other studies [6, 11] and might be explained by selective survival. Also, it is important to consider an effect of aging, such that experiences and/or perceptions of discrimination may change across life course, suggesting the importance of considering the specific cohort and period context in which discrimination was measured [6, 13].

Regarding racial disparities, our study demonstrated that reports of discriminatory experiences were respectively 57, 49 and 20% higher among yellow/indigenous, blacks and browns, in comparison to whites. As yellows and indigenous constitute a minority group, also in numeric terms, researchers usually exclude these individuals from analysis due to the small sample size. To our knowledge, it was the first time a representative study investigated discrimination among yellow and indigenous older adults in Brazil. The higher levels of perceived discrimination we found among indigenous and blacks are consistent with the marks that slavery left in Brazil, regarding the social position of these individuals throughout successive generations. Aspects such as greater exposure to early mortality along with social disorganization and migration to cities, living in poor conditions, certainly have contributed to perpetuate the socioeconomic marginality of these groups [30]. Lifetime socioeconomic differences across successive generations have been identified as the main cause of racial inequality in health [4, 30]. It has been suggested that perceived discrimination and its impact on health play a central role in the origin of these inequalities [4, 30]. Some authors also argue that racial discrimination in Brazil is mostly notable in “hard domains”, such as job market, affective-sexual relationships, and the interactions with the police [3]. Under this perspective, discrimination in “hard domains” would occur as an expression of dominance and oppression, viewed as a struggle for power and privilege [4]. In other contexts, termed “soft domains”, race would tend to seem irrelevant for social relations and interpersonal contact. These domains include both leisure places and activities, such as pubs, having a talk with the neighbor, samba, carnival, and locals of spiritual or religious manifestations [3]. It might explain our findings of racial disparities on perceived discrimination in seeking health care, whereas health care settings as “hard domains”. However, we found significant racial disparities on perceived discrimination in social gatherings – understood as a “soft domain”, and no disparities in the work place - a “hard domain”. It seems plausible that the prevalence of perceived discrimination in the work place may decrease and no disparities among racial groups be observed, as individuals get older and leave the job market. From now, it is necessary to investigate the relationship between racial disparities and specific domains of perceived discrimination among older adults along with how disparities affect health outcomes. A deeper understanding on these topics did not constitute our objectives in this study.

According to our results, the more educated participants perceived more discrimination. Those with higher educational attainment may be more aware of discriminatory treatment or more likely to identify it as such, or may engage in activities and move through social environments with a more diverse set of individuals, placing them at greater exposure (out of place) [13]. Unexpectedly, no association was observed with household wealth, which might potentially protect individuals from exposure to situations that give rise to discrimination and provide a greater sense of control or security [31]. Some authors identified level of education and wealth as the most significant correlates of perceived discrimination, regardless of the discriminatory situation itself [10]. Considering reports of everyday discrimination among older adults, findings are quite heterogeneous concerning educational level. Some studies showed that the participants who reported having experienced discrimination were more likely to be more educated [10, 31] and less wealthy [10, 27, 31]. However, there are no significant differences regarding educational attainment for other samples [27, 31]. Additionally, a Brazilian regional study including young, middle and older adults, showed no association between perceived any discrimination and both educational attainment and household wealth [11]. In agreement with this previous study, we also observed that participants in poor health status and low social trust have consistently higher prevalence of reporting discrimination. Although we cannot establish causal relationships linking discrimination and poorer health status, there is considerable evidence on this association [4, 5]. There is also consistent evidence that social support may moderate the effects of discrimination on health. However, our study design does not allow us to investigate if people have low trust due to experiences of discrimination or, whether people who have less trust are more likely to interpret some actions as discriminatory or to report experiences of discrimination more often. Regarding the longitudinal perspective of ELSI-Brazil, we will be able to explore the complex relationships between discrimination, poor health and low social trust among older adults in a near future.

It is also noteworthy that about one in ten participants reported perceived discrimination in seeking health care. This finding is quite consistent with both international and national literature [10, 12, 31]. Discrimination may be evident in how clinical staff communicate with older patients and in the quality of care they provide. It can affect physicians’ behaviors and their decision-making, contributing to disparities in health care. For patients with major chronic conditions, such as older adults, discriminatory experiences may reduce engagement with the medical care system, affecting health promotion and disease management [32]. Whereas that most Brazilian older adults depends exclusively of the national health system, the *Sistema Único de Saúde* or SUS, our finding shows a very concerning trending of the healthcare in the country. The SUS provides comprehensive and universal care through decentralized management and provision of health services that are free of charge at the point of delivery [14]. Universality, comprehensiveness and equity constitute the cornerstones of the system, which includes that all citizens must be treated respectfully and appropriately by health care professionals, regardless any personal attribute. Future research may provide better understanding on perceived discrimination in healthcare settings among Brazilian older adults.

Our study is not without limitations. First, the true magnitude of rural Brazil has been discussed [16, 17]. The current methodology employed by IBGE establishes by means of municipal laws the limits of urban areas and classifies as rural those areas located outside these boundaries. If characteristics such as size, density, diversity and complexity define urban areas [18], it is noteworthy that many Brazilian municipalities currently classified as urban do not present these core aspects [16, 17]. A new urban-rural classification, aligned to international community, has been proposed by IBGE and will be incorporated to the 2020 Population Census. Based on three criteria - population size, demographic density, and localization in relation to the main urban centers - this new typology found 76% of the Brazilian population as predominantly urban, which corresponds to 26% of the total municipalities [17]. Regardless this potential limitation, our study took into account the official classification employed in the country. Second, our research used a generic measure of discrimination. Unfortunately, our data did not allow participants to attribute discrimination to one or more possible causes, such as gender, race, age, weight, physical disability, or other. Despite attribute’s relevance, many studies indicate that the experience of unfairness or mistreatment may be more important for health than what the mistreatment is attribute to [6]. Moreover, some authors argue that focuses on a single attribution for discriminatory experiences may ignore that individuals often occupy more than one socially disadvantaged status and these status may interact to shape their experiences, which is termed intersectionality. We do believe that our multivariate analysis partially offset this limitation, as we controlled for characteristics typically related to discrimination, such as age, gender, and SES. Third, this study may be subjected to bias, once place of residence might play a role on either minimization or vigilance bias. Minimization bias occurs when individuals perceive less discrimination than actually exists. Subtle ways of discrimination related to ambiguous situations could lead to minimization bias. It also might occur when individuals who belong to disadvantaged groups internalize unfair treatment as a natural or normal phenomenon [3, 6]. In our analysis, rural older adults might be subjects to this type of bias. On the other hand, their urban counterparts might perceive more discrimination than actually exists, which is known as vigilance bias. Fourth, our study captured the perspective of individuals who reported discrimination. Another way to investigate it could consider the perspective of the stigmatizing individuals, which is termed actual discrimination (e.g. attitudes from health professionals towards groups with specific personal attributes). Together, both perspectives would show whether and to what extent perceived and actual discrimination effectively corresponds. Since we measured perceived discrimination, we cannot draw conclusions about levels of actual discrimination.

Our study also has important strengths. Using a national source of high quality information on individual aged 50 years or more, to our knowledge we carried out the first national study on perceived discrimination among older adults. Besides, it included participants commonly excluded from analyses: yellows, indigenous and rural dwellers. Due to the design of ELSI-Brazil, we will be able to provide longitudinal information on reports of discrimination in a near future.

## Conclusions

In brief, our study explored the prevalence and correlates of perceived discrimination among a representative sample of Brazilian older adults. Our main findings show that: (1) the urban environment plays a core role in perceived discrimination; (2) yellows and indigenous are more likely to report discriminatory experiences, followed by blacks; (3) health care settings constitute the most common domain where discriminatory experiences occurred. Our findings may contribute to fulfill the knowledge gap on discrimination among older adults living in developing countries.

## Additional file

Additional file 1: Principal component analysis (PCA) on health status. A composite measure of the health status was obtained, based on PCA. The selected variables included self-rated health, number of the last 30 days spent in poor mental and/or physical health, and history of medical diagnosis from a list of 12 prevalent chronic diseases/conditions, such as hypertension, diabetes, depression and arthritis. The resulting health status score was divided into tertiles, representing individual with few health problems, some health problems and many health problems. (PDF 163 kb)

## Abbreviations

CI: Confidence Interval
ELSA: English Longitudinal Study of Ageing
ELSI: Brazilian Longitudinal Study of Aging (*Estudo Longitudinal da Saúde dos Idosos Brasileiros)*
FIOCRUZ: Oswaldo Cruz Foundation *(Fundação Oswaldo Cruz)*
HRS: Health and Retirement Study
IBGE: Brazilian Institute of Geography and Statistics *(Instituto Brasileiro de Geografia e Estatística)*
PCA: Principal Components Analysis
PR: Prevalence Ratios
SES: Socioeconomic status
SUS: Unified Health System *(Sistema Único de Saúde)*

## References

[1] Kessler RC, Mickelson KD, Williams DR (1999). The prevalence, distribution, and mental health correlates of perceived discrimination in the United States. J Health Soc Behav.

[2] Ayalon L, Gum AM. The relationships between major lifetime discrimination, everyday discrimination, and mental health in three racial and ethnic groups of older adults. Aging Ment Health. 2011; 15: 587–594. doi: 10.1080/13607863.2010.543664. 10.1080/13607863.2010.543664 21815851

[3] Bastos JL, Faerstein E. Discriminação e saúde: perspectivas e métodos. Rio de Janeiro, RJ: Editora FIOCRUZ; 2012.

[4] Krieger N. Discrimination and health inequities. In: Berkman F, Kawachi I, Glymour MM. Social epidemiology. 2nd ed. New York, NY: Oxford University Press; 2014.

[5] Pascoe EA, Richman LS (2009). Perceived discrimination and health: a meta-analytic review. Psychol Bull.

[6] Lewis TT, Cogburn CD, Williams DR (2015). Self-reported experiences of discrimination and health: scientific advances, ongoing controversies, and emerging issues. Annu Rev Clin Psycho.

[7] Levy BR, Slade MD, Murphy TE, Gill TM (2012). Association between positive age stereotypes and recovery from disability in older persons. JAMA..

[8] Levy BR, Zonderman AB, Slade MD, Ferrucci L (2012). Memory shaped by age stereotypes over time. J Gerontol B Psychol Sci Soc Sci.

[9] Sutin AR, Stephan Y, Carretta H, Terracciano A (2015). Perceived discrimination and physical, cognitive, and emotional health in older adulthood. Am J Geriatr Psychiatry.

[10] Rippon I, Kneale D, de Oliveira C, Demakakos P, Steptoe A (2014). Perceived age discrimination in older adults. Age Ageing.

[11] Macinko J, Mullachery P, Proietti FA, Lima-Costa MF (2012). Who experiences discrimination in Brazil? Evidence from a large metropolitan region. Int J Equity Health.

[12] Boccolini CS, Boccolini PMM, Damacena GN, Ferreira APS, Szwarcwald CL. Factors associated with perceived discrimination in health services of Brazil: Results of the Brazilian National Health Survey, 2013 Cien Saude Colet 2016; 21(2): 371–378. doi: 10.1590/1413-81232015212.19412015. 10.1590/1413-81232015212.19412015 26910145

[13] Burgard S, Castiglione DP, Lin KY (2017). Differential reporting of discriminatory experiences in Brazil and the United States. Cad. Saúde Pública..

[14] Paim J, Travassos C, Almeida C, Bahia L, Macinko J (2011). The Brazilian health system: history, advances and challenges. Lancet.

[15] Instituto Brasileiro de Geografia e Estatística (IBGE). Sistema IBGE de Recuperação Automática (SIDRA) / Censo Demográfico 2010. https://sidra.ibge.gov.br/tabela/2093. Acessed 8 Jan. 2018.

[16] Wanderley MNB, Favareto A. A singularidade do rural brasileiro: implicacoes para as tipologias territoriais e a elaboracao de politicas publicas. In: Miranda C, Silva H (org). Concepcoes da ruralidade contemporanea: as singularidades brasileiras. Brasilia: Instituto Interamericano de Cooperacao para a Agricultura (IICA); 2013.

[17] Instituto Brasileiro de Geografia e Estatística (IBGE) (2017). Classificação e caracterização dos espaços rurais e urbanos no Brasil: uma primeira aproximação. Rio de Janeiro. RJ: IBGE.

[18] Vlahov D. A pivotal moment for urban health. Cad.Saúde Pública. 2015; 31 Sup: S7-S8. doi: 10.1590/0102-311XPE01S115 10.1590/0102-311XPE01S115 26648352

[19] Caiaffa WT, Ferreira FR, Ferreira AD, Oliveira CL, Camargos VP, Proietti FA (2008). Urban health: "the city is a strange lady, smiling today, devouring you tomorrow". Ciênc Saúde Coletiva.

[20] Diez Roux AV. Health in cities: is a systems approach needed? CadSaúde Pública 2015; 31 Sup: S9-S23. doi: 10.1590/0102-311XDE01S115 10.1590/0102-311XDE01S115 26648353

[21] Ezeh A, Oyebode O, Satterthwaite D (2017). The history, geography, and sociology of slums and the health problems of people who live in slums. Lancet.

[22] Lima-Costa MF, Andrade FB, Souza PRB Jr, et al. The Brazilian longitudinal study of aging (ELSI-Brasil): objectives and design. Am J Epidemiol. 2018; Jan 31. 10.1093/aje/kwx387. 10.1093/aje/kwx387 PMC6031009 29394304

[23] Instituto Brasileiro de Geografia e Estatística (IBGE). Desenho conceitual. In: __ Metodologia do Censo Demográfico 2010. 2ª ed. Rio de Janeiro, RJ: IBGE; 2016:277–351.

[24] Ismail K (2008). Unravelling factor analysis. Evid Based Ment Health.

[25] Robbins AS, Chao SY, Fonseca VP (2002). What’s the relative risk? A method to directly estimate risk ratios in cohort studies of common outcomes. Ann Epidemiol.

[26] StataCorp: Stata user’s guide. Release 12. College Station, TX: Stata corporation; 2011.

[27] Shankar A, Hinds P (2017). Perceived discrimination: associations with physical and cognitive function in older adults. Health Psychol.

[28] World Bank. Data Bank: Poverty and Equity database. Gini index (World Bank Estimate). Available at: https://data.worldbank.org/country/brazil. Accessed 8 Jan 2018.

[29] Travassos C, Viacava F (2007). Access to and use of health services by rural elderly, Brazil, 1998 and 2003. Cad.Saúde Pública..

[30] Chor D, Lima CRA (2005). Epidemiologic aspects of racial inequalities in health in Brazil. Cad Saúde Pública.

[31] Rippon I, Zaninotto P, Steptoe A (2015). Greater perceived age discrimination in England than the United States: results from HRS and ELSA. J Gerontol B Psychol Sci Soc Sci.

[32] Nguyen TT, Vable AM, Glymour MM, Nuru-Jeter A. Trends for reported discrimination in health care in a national sample of older adults with chronic conditions. J Gen Intern Med. 2017;15. 10.1007/s11606-017-4209-5. 10.1007/s11606-017-4209-5 PMC5834956 29247435

