# Supplementary material for: Perceived discrimination among older adults living in urban and rural areas in Brazil: a national study (ELSI-Brazil)
Source: BMC Geriatr. 2019 Mar 4;19:67. doi: 10.1186/s12877-019-1076-4 (PMC6399885; doi:10.1186/s12877-019-1076-4)
Supplement: Supplementary file 1 — Principal component analysis (PCA) on health status. A composite measure of the health status was obtained, based on PCA. The selected variables included self-rated health, number of the last 30 days spent in poor mental and/or physical health, and history of medical diagnosis from a list of 12 prevalent chronic diseases/conditions, such as hypertension, diabetes, depression and arthritis. The resulting health status score was divided into tertiles, representing individual with few health problems, some health problems and many health problems. (PDF 163 kb) [file 12877_2019_1076_MOESM1_ESM.pdf]

ADDITIONAL FILE 1

Principal component analysis on health status.

Component	Eigenvalue	Difference	Proportion	Cumulative
Comp1	2,5503	1,2515	0,1962	0,1962
Comp2	1,2987	0,0109	0,0999	0,2961
Comp3	1,2879	0,1981	0,0991	0,3951
Comp4	1,0898	0,1140	0,0838	0,4790
Comp5	0,9757	0,0838	0,0751	0,5540
Comp6	0,8919	0,0410	0,0686	0,6226
Comp7	0,8510	0,0191	0,0655	0,6881
Comp8	0,8318	0,1032	0,0640	0,7521
Comp9	0,7286	0,0479	0,0560	0,8081
Comp10	0,6807	0,0397	0,0524	0,8605
Comp11	0,6410	0,0120	0,0493	0,9098
Comp12	0,6290	0,0854	0,0484	0,9582
Comp13	0,5436	.	0,0418	1,0000

Variable	Comp1	Comp2	Comp3	Comp4	Comp5	Comp6	Comp7	Comp8	Comp9	Comp10	Comp11	Comp12	Comp13
DCV	0,2171	0,0698	0,3559	0,2304	0,3216	0,2592	-0,4565	-0,5828	-0,0658	0,0878	-0,0049	0,1872	0,0496
Hypertension	0,1838	-0,0489	0,4445	0,2741	0,0521	0,1655	-0,2784	0,7507	-0,0381	-0,0442	0,1019	-0,0448	0,0644
Diabetes	0,1022	-0,0348	0,3924	0,2520	-0,6336	0,2911	0,4685	-0,2110	0,0759	-0,0459	0,0677	0,0378	0,0821
Stroke	0,1249	-0,0303	0,4467	0,0761	0,4078	-0,6309	0,4333	-0,0562	0,1327	-0,0263	0,0398	0,0251	0,0485
Asthma	0,1490	0,6822	-0,0601	-0,0188	-0,1016	-0,0716	0,0468	0,1736	0,1077	0,1295	-0,2287	0,6159	-0,0247
COPD	0,1770	0,6773	-0,0142	-0,0030	-0,0104	-0,0275	-0,0084	-0,0796	-0,0833	-0,1098	0,2920	-0,6274	0,0675
Arthritis	0,3272	-0,0735	-0,3308	0,3291	0,0698	-0,0399	0,0924	-0,0208	-0,3008	-0,7178	0,0587	0,2097	-0,0368
Ostheoporosis	0,3040	-0,0841	-0,2946	0,4177	0,0828	-0,0023	0,2364	0,0493	-0,3871	0,6352	-0,0849	-0,1133	-0,0274
Back pain	0,3453	-0,1152	-0,2887	0,1967	0,0101	0,0132	-0,0763	-0,0083	0,7761	0,0889	0,3126	-0,0122	-0,1766
Depression	0,3177	-0,0168	-0,0136	-0,2974	0,3575	0,4868	0,3275	0,0704	0,1857	-0,1127	-0,4782	-0,1777	0,1604
Self-rated health	0,3994	-0,0841	0,1683	-0,1926	-0,2909	-0,2411	-0,2071	-0,0474	-0,0757	-0,0382	-0,3878	-0,1876	-0,6223
Physical health	0,3968	-0,1594	-0,0766	-0,2148	-0,2959	-0,3077	-0,2525	-0,0365	-0,0011	0,0483	-0,0804	0,0002	0,7135
Mental health	0,3207	-0,0897	0,0671	-0,5559	0,0621	0,1586	0,1430	0,0345	-0,2636	0,1251	0,5932	0,2532	-0,1541

DCV: cardiovascular disease (heart attack, angina pectoris, heart failure); COPD: chronic obstructive pulmonary disease.

Variable	Kaiser-Meyer-Olkin Test
DCV	0,7640
Hypertension	0,7362
Diabetes	0,6750
Stroke	0,7152
Asthma	0,5623
COPD	0,5820
Arthritis	0,7677
Ostheoporosis	0,7626
Back pain	0,7909
Depression	0,7669
Self-rated health	0,7504
Physical health	0,7388
Mental health	0,7456
**Overall**	**0,7310**

DCV: cardiovascular disease (heart attack, angina pectoris, heart failure); COPD: chronic obstructive pulmonary disease.
